# Mechanical Synchronization of MEMS Electrostatically Driven Coupled Beam Filters

**DOI:** 10.3390/mi12101191

**Published:** 2021-09-30

**Authors:** Richard Syms, Adam Bouchaala

**Affiliations:** Department of Electrical and Electronic Engineering, Imperial College London, Exhibition Road, London SW7 2AZ, UK; a.bouchaala@imperial.ac.uk

**Keywords:** mechanical filter, coupled resonator, MEMS

## Abstract

Micro-electromechanical systems (MEMS) bandpass filters based on arrays of electrostatically driven coupled beams have been demonstrated at MHz frequencies. High performance follows from the high Q-factor of mechanical resonators, and electrostatic transduction allows tuning, matching and actuation. For high-order filters, there is a conflict between the transduction mechanism and the coupling arrangement needed for dynamic synchronization: it is not possible to achieve synchronization and tuning simultaneously using a single voltage. Here we propose a general solution, based on the addition of mass-loaded beams at the ends of the array. These beams deflect for direct current (DC) voltages, and therefore allow electrostatic tuning, but do not respond to in-band alternating current (AC) voltages and hence do not interfere with synchronization. Spurious modes generated by these beams may be damped, leaving a good approximation to the desired response. The approach is introduced using a lumped element model and verified using stiffness matrix and finite element models for in-plane arrays with parallel plate drives and shown to be tolerant of the exact mass value. The principle may allow compensation of fabrication-induced variations in complex filters.

## 1. Introduction

Because of their intrinsically high Q-factor, electrical filters based on mechanical resonators have long been of interest for signal processing [1,2,3,4,5,6]. Most are bandpass filters based on arrays of coupled resonators. Miniaturization using micro-electro-mechanical systems (MEMS) technology allowed operating frequencies to be raised and the fundamental mechanisms limiting Q-factors (gas damping and thermoelastic friction) to be understood [7,8,9,10,11,12]. MEMS filters were initially demonstrated at high kHz frequencies as lumped-element systems driven by electrostatic comb-drives [13,14,15,16,17] and then at MHz frequencies as coupled-beam arrays driven by parallel-plate actuators [15,18,19,20,21,22,23,24,25]. Matching [20], tuning [26,27,28,29,30] and coupling [31,32,33,34,35] can all be performed electrostatically. Alternative designs based on large linear arrays, rings, and 2D and 3D arrays have all been proposed to achieve different filter functionalities [36,37,38,39,40,41,42].

Several difficulties remain. Coupling must be weak to achieve a bandwidth suitable for applications such as intermediate frequency (IF) filtering. Mechanical coupling elements must then be nanostructured [43,44,45,46]. Electrode gaps must also be very small for realistic input and output impedance, further complicating fabrication [47,48,49,50]. However, a more fundamental difficulty exists. In a system of two mechanically coupled beams, the modified resonant frequencies of the two beams are inherently matched, because the effects of the coupling springs on each beam are identical. However, in high-order filters, which require more than two beams, the inner beams experience the stiffness modification of a different number of coupling springs to the outer beams. The suspension needed to ensure equal DC deflection (and hence equal electrostatic tuning) then no longer yields a synchronous AC response. Electrostatic stiffness modification is thus incompatible with the dynamic synchronization needed for correct collective operation of the array. The effect can be compensated by applying different DC tuning voltages to the inner and outer beams [17]. Unfortunately, it is difficult to establish the voltages needed even in simulation, using either the finite element method (FEM) [51,52,53], which involves accurate results using lengthy run times, or the stiffness matrix method (SMM) [54,55,56,57], which provides approximate results very quickly. The need for multiple DC voltages may present even more problems in production if tuning is needed to compensate for fabrication variations.

Here we propose a simple method of eliminating the second voltage, using a mechanical modification that separates the problems of DC tuning and obtaining a synchronized AC response. Additional resonators are introduced at either end of the array, together with the coupling elements needed to obtain the correct DC response. These resonators are mass-loaded so their resonances lie far enough from those of the original array that they do not take part in collective oscillation. The additional modes may be damped, leaving the filter response to be determined by the original array, which is now properly synchronized. The solution is illustrated in terms of arrays with in-plane motion and driven by parallel plate actuators. The solution is general but is illustrated in terms of arrays with in-plane motion and driven by parallel plate actuators. The design problem is introduced through FEM simulations in Section 2, and a solution is proposed. The principle is demonstrated in Section 3 using a lumped element model (LEM), and performance is verified in Section 4 using the SMM. Conclusions are drawn in Section 5.

## 2. Design Problem for Coupled Beam Arrays

In this Section, we describe the contradiction inherent in electrostatic synchronization and present the argument for dynamic compensation using mass-loaded beams.

### 2.1. Electrostatic Synchronization

**Figure 1:**
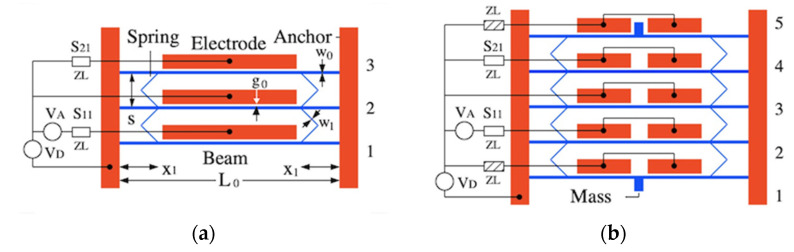
Band-pass coupled-beam filters: (**a**) 3-beam system and (**b**) 5-beam system with compensating springs, mass-loaded end beams and split electrodes. Red—fixed parts; blue—movable parts.

Figure 1a shows a 3-beam in-plane coupled beam array configured as a band-pass filter. Here red and blue features show fixed and undercut moving parts, and connections are shown as wires. Each horizontal element is a built-in MEMS beam. Adjacent beams are connected near their roots by two sets of weak nanostructured meander springs, placed symmetrically. These springs couple the beams together but also upshift their resonant frequency. The AC input $V_{A}$ and output are connected to parallel plate transducers at beams 1 and 3 via loads $z_{L}$, and scattering parameters $S_{11}$ and $S_{21}$ describe reflection and transmission at these ports. An additional DC voltage $V_{D}$ is used to linearize the input and obtain an output, and to allow electrostatic tuning. To ensure synchronization, the central beam is also equipped with a transducer. Application of the same DC voltage to each transducer must then cause equal deflection of each beam, and equal tuning.

Unfortunately, this arrangement does not provide dynamic synchronization. If each beam vibrates in isolation, with the others stationary, the end beams must have the elastic support of one pair of springs, while the central beam has that of two. Consequently, the effective resonant frequencies of beams 1 and 3 must differ from that of beam 2. One solution might be to connect an additional pair of springs from the end beams to anchors, restoring dynamic synchronization. However, application of the same DC voltage to each transducer cannot then cause equal beam motion since this would require deflection of the end springs alone. A common approach to such problems is to apply a different DC bias to each transducer [17,25]. However, the correction is extremely tedious.

### 2.2. FEM Simulation

The problem can be illustrated with FEM simulations of the device layout in Figure 1a, carried out using the commercial software COMSOL^®^ [58]. Three coupled modules (Solid Mechanics, Electrostatics and Electrical Circuit) were used. First, the mechanical layout and constraints were set up, and elastic and inertial constants were defined. Inertial damping was estimated from the Q-factor. Electrostatic drives were defined on opposing surfaces of cuboid air volumes between each beam and a fixed electrode. Terminals were added to allow application of DC and AC voltages, with the AC input and output being connected via load resistors $z_{L}$. The mechanism and air gaps were meshed using a free triangular mesh, using different mesh sizes to reduce simulation time. A frequency sweep was used to calculate S-parameter variations from terminal currents.

The following values were assumed for dimensions: $L_{0}=150\;\mu m$, $w_{0}=3\;\mu m$, $d_{0}=4\;\mu m$, $\alpha =x_{1}/L_{0}=0.25$, s=6 μm, $w_{1}=0.1\;\mu m$, and $g_{0}=0.1\;\mu m$. A density of $\rho =2332\;\mathrm{kg}/m^{3}$, Young’s moduli of $E_{0}=169\times 10^{9\;}N/m^{2}$ and $E_{1}=130\times 10^{9}N/m^{2}$ and a Poisson’s ratio of ν=0.28 were chosen to model devices in (100) Si with the micro- and nano-structured beams in the <110> and <010> directions [59]. A quality factor of Q=5000 was taken as representative; however, its value is of limited significance provided it is large. An AC voltage of $V_{A}=0.1\;\mathrm{mV}$ was used for dynamic actuation.

**Figure 2:**
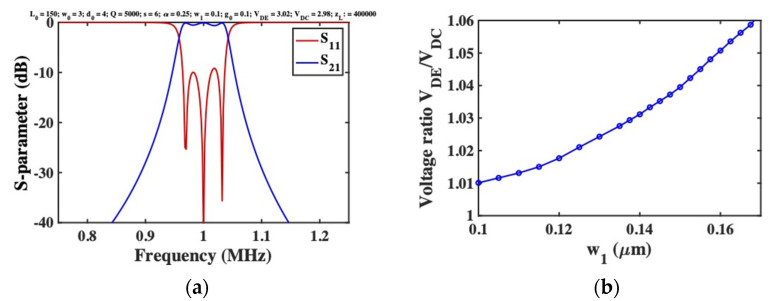
**(a**) Frequency response of 3-beam filter with purely electrostatic tuning, as predicted by the FEM; (**b**) extracted variation of voltage ratio $V_{DE}/V_{DC}$ with linking spring width $w_{1}$.

DC voltages $V_{DE}$ and $V_{DC}$ were first applied to the end and central beams to achieve tuning at a design frequency of $f_{1}=1$, as $V_{DE}=3.02\;V$ and $V_{DC}=2.98\;V$, below the snap-down voltage of ~3.5 V. The impedance $z_{L}$ was then adjusted to achieve matching, as $z_{L}=400\;k\Omega$. Figure 2a shows the resulting variation of the S-parameters with frequency. Apart from the high value of $z_{L}$ (a known feature of coupled beam devices [21,47]), high performance is obtained; the response is bandpass, with correct tuning and broadband matching. However, determination of the DC voltages is a laborious process, which must be repeated each time parameters change. For example, Figure 2b shows the extracted variation of $V_{DE}/V_{DC}$ with the width $w_{1}$ of the linking springs, likely to vary in production. The ratio is not constant, implying that both voltages must be continually rediscovered. Similar effects occur if the electrode gap $g_{0}$ is altered, and even minor departure from suitable voltages leads to an unrecognisable response or failed simulation.

### 2.3. Dynamic Synchronization

Here we propose the solution shown in Figure 1b, which allows a single DC voltage. The array is now equipped with an additional beam at either end, coupled to its neighbour by springs and equipped with transducers to which DC voltages are applied. As in Figure 1a, a common DC voltage will yield equal deflections of all 5 beams. The additional beams are mass-loaded at their midpoint. Ideally, this mass will be formed by a variation in layout. However, care will be required to locate it in the available space without modifying beam stiffness. One possibility, shown here, is to place both masses outside the array, splitting the upper electrode to clear the mass (and splitting the remainder to retain synchronization). If the masses are large enough, the resonances of beams 1 and 5 will differ sufficiently from that of beams 2–4 that they take no part in the in-band response. Thus, the array may be considered as five statically synchronized beams for DC bias, and three dynamically synchronized beams for AC signals.

## 3. Lumped Element Model

In this section we construct a lumped element model of a set of coupled resonant beams with parallel-plate drives and show how mass-loading can control the response.

### 3.1. Resonant Modes of a Vibrating Beam

We start by considering the resonant modes of an undamped, undriven beam of length $L_{0}$, width $w_{0}$ and depth d, formed in a material of density ρ and Young’s modulus $E_{0}$. These are the solutions to the dynamic Euler beam-bending equation for a clamped-clamped beam, namely [60]:

(1) $$\Upsilon_{\nu }\left(x\right)=\left(\frac{\gamma }{\sqrt{L_{0}}}\right)\left\{\frac{sin\left(\beta_{\nu }x\right)-sinh\left(\beta_{\nu }x\right)}{sin\left(\beta_{\nu }L_{0}\right)-sinh\left(\beta_{\nu }L_{0}\right)}-\frac{cos\left(\beta_{\nu }x\right)-cosh\left(\beta_{\nu }x\right)}{cos\left(\beta_{\nu }L_{0}\right)-cosh\left(\beta_{\nu }L_{0}\right)}\right\},$$

Here $\Upsilon_{\nu }\left(x\right)$ is transverse displacement, and x is position along the beam. The eigenvalues $\beta_{\nu }$ are related to the angular resonant frequencies $\omega_{\nu }$ by $\beta_{\nu }^{4}=\omega_{\nu }\rho A_{0}/E_{0}I_{0}$, where $A_{0}=w_{0}d$ is the area and $I_{0}=w_{0}^{3}d$/12 is the second moment of area, and satisfy the characteristic equation $\cos\left(\beta_{\nu }L_{0}\right)\cosh\left(\beta_{\nu }L_{0}\right)=1$. This equation has a set of discrete solutions. Here we are concerned with the lowest-order mode, which corresponds to $\beta_{1}L_{0}=\sqrt{22.37}$. The term γ is a normalization constant, chosen so that $\int_{0}^{L_{0}}\Upsilon_{\nu }{}^{2}dx=1$. More generally, the beam may have a distributed damping r per unit length and be driven by a force f per unit length.

### 3.2. Lumped Element Model

**Figure 3:**
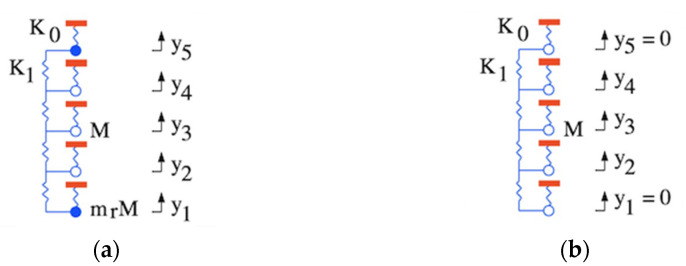
Lumped models of Figure 1b, with (**a**) mass loaded and (**b**) fixed end elements.

Figure 3a shows the lumped element model, in which each resonator except the first and the last is considered as a mass M supported on a spring of stiffness $K_{0}$. Again, the red and blue features show fixed and moving parts, and damping is omitted for clarity.

Equivalence with the distributed model is established using the factors $\eta_{1}=\mathrm{avg}\left(\Upsilon_{1}\right)/\max\left(\Upsilon_{1}\right)$ and $\eta_{1}=\mathrm{avg}\left(\Upsilon_{1}^{2}\right)/\max\left(\Upsilon_{1}^{2}\right)$ which have the values 0.5232 and 0.3965, respectively. These allow the lumped mass M, stiffness $K_{0}$, damping coefficient R and force F to be found as [21]:

(2) $$M=\rho A_{0}L_{0}\eta_{2},\;K_{0}=\omega_{1}^{2}M,\;\;R=rL_{0}\eta_{2},\;F=fL_{0}\eta_{1},$$

For the outer elements, the mass is increased to $m_{r}M$, where $m_{r}=\left(M+\Delta M\right)/M$ is a mass ratio and ΔM is an additional lumped-element mass. The coupling springs are formed from elements of length $L_{1}$, width $w_{1}$, depth d, density ρ and Young’s modulus $E_{1}$, inclined at 45° angles to give a separation $s=L_{1}\sqrt{2}$ between the beams. The equivalent spring constant and mass of each pair are $k_{1}=24E_{1}I_{1}/L_{1}^{3}$ and $m_{1}=2\rho A_{1}L_{1}$, where $I_{1}=w_{1}^{3}d/12$ and $A_{1}=w_{1}d$. Here a different elastic modulus $E_{1}$ is introduced as before, and the mass $m_{1}$ is half the actual mass, to model the motion of the centre of mass of each spring. Perturbation theory then allows an equivalent lumped element coupling stiffness $K_{1}$ to be found as:

(3) $$K_{1}=\left(k_{1}-\omega_{1}^{2}m_{1}\right)L_{0}\Upsilon_{1}^{2}\left(x_{1}\right)\eta_{2},$$

For very small springs, the effect of the mass $m_{1}$ may often be ignored. Development of a model for electrostatic transducers is more complicated. Following [21] we assume that the electrodes act as parallel plate capacitors with capacitance:

(4) $$C=\varepsilon_{0}L_{0}d/\left(g_{0}-y_{D}\right),$$

Here $g_{0}$ is the initial gap and $y_{D}$ is a static displacement. Application of a DC voltage $V_{D}$ generates a static force:

(5) $$F_{D}=\frac{1}{2}C^{\prime }V_{D}^{2}\eta_{1},$$

Here $C^{\prime }=\varepsilon_{0}L_{0}d/{\left(g_{0}-y_{D}\right)}^{2}$ is the derivative of C. Static equilibrium then implies that $F_{D}=K_{0e}y_{D}$. Here $K_{0e}$ is the effective stiffness, which may reasonably be approximated as $K_{0}$. This is a standard snap-down problem, leading to the cubic equation:

(6) $$y_{nD}^{3}-2y_{nD}^{2}+y_{nD}-\gamma =0,$$

Here $y_{nD}=y_{D}/g_{0}$ is the normalised deflection and $\gamma =\varepsilon_{0}L_{0}dV_{D}^{2}\eta_{1}/\left(2K_{0}g_{0}^{3}\right)$. Solution allows calculation of C, $C^{\prime }$ and the second derivative $C^{\prime \prime }$. When an additional AC voltage $V_{A}$ is applied from a source with output impedance $z_{L}$, the result is an AC force $F_{A}$, a reduction in stiffness ΔK and an effective load $Z_{L}$ given by:

(7) $$F_{A}=V_{D}C^{\prime }\eta_{1}V_{A},\;\Delta K=\frac{1}{2}V_{D}^{2}C^{\prime \prime }\eta_{2},Z_{L}={\left(V_{D}C^{\prime }\eta_{1}\right)}^{2}z_{L},$$

In general, the characteristic impedance $Z_{0}$ of a coupled beam array is complex, but for an infinite lossless array at resonance it has the real value:

(8) $$Z_{0R}=K_{1}/\omega_{1e},$$

Here $\omega_{1e}=\surd \{(K_{0}+\frac{2K_{1}}{M}\}$ is the effective angular resonant frequency. Matching can then be achieved by choosing $Z_{L}=Z_{0R}$. This requires the load resistance $z_{L}$ to be chosen so that $K_{v}^{2}z_{L}=Z_{0R}$. However, large values of $z_{L}$ are needed if $K_{v}$ is small [21].

### 3.3. Coupled Equations

Combining the results above it is simple to show that the governing equations for a 5-element array with input and output ports at n=2 and n=4 subject to a harmonic drive $F_{A}=F_{0}\exp\left(j\omega t\right)$ at angular frequency ω are:

$$\left\{\left(K_{0}+K_{1}-\Delta K\right)-m_{r}M\omega^{2}+j\omega R\right\}y_{1}-K_{1}y_{2}=0$$

$$\left\{\left(K_{0}+2K_{1}-\Delta K\right)-M\omega^{2}+j\omega \left(R+Z_{L}\right)\right\}y_{2}-K_{1}\left(y_{1}+y_{3}\right)=F_{0}$$

$$\left\{\left(K_{0}+2K_{1}-\Delta K\right)-M\omega^{2}+j\omega R\right\}y_{3}-K_{1}\left(y_{2}+y_{4}\right)=0$$

$$\left\{\left(K_{0}+2K_{1}-\Delta K\right)-M\omega^{2}+j\omega \left(R+Z_{L}\right)\right\}y_{4}-K_{1}\left(y_{3}+y_{5}\right)=0$$

(9) $$\left\{\left(K_{0}+K_{1}-\Delta K\right)-m_{r}M\omega^{2}+j\omega R\right\}y_{5}-K_{1}y_{4}=0$$

These equations can be solved by inversion of the equivalent matrix representation, and reflection and transmission scattering parameters can then be found using standard methods. Here, however, we focus on the resonant modes.

### 3.4. Resonant Modes

In the absence of damping, loading and a driving force, Equation (9) reduce to:

$$\left\{\left(K_{0}+K_{1}-\Delta K\right)-m_{r}M\omega^{2}\right\}y_{1}-K_{1}y_{2}=0$$

$$\left\{\left(K_{0}+2K_{1}-\Delta K\right)-M\omega^{2}\right\}y_{2}-K_{1}\left(y_{1}+y_{3}\right)=0$$

$$\left\{\left(K_{0}+2K_{1}-\Delta K\right)-M\omega^{2}\right\}y_{3}-K_{1}\left(y_{2}+y_{4}\right)=0$$

$$\left\{\left(K_{0}+2K_{1}-\Delta K\right)-M\omega^{2}\right\}y_{4}-K_{1}\left(y_{3}+y_{5}\right)=0$$

(10) $$\left\{\left(K_{0}+K_{1}-\Delta K\right)-m_{r}M\omega^{2}\right\}y_{5}-K_{1}y_{4}=0$$

Introducing the terms $\omega_{1e}^{2}=\left(K_{0}+2K_{1}-\Delta K\right)/M$, $\omega_{1m}^{2}=\left(K_{0}+K_{1}-\Delta K\right)/m_{r}M$, $\kappa =K_{1}/M$ and $\kappa_{m}=K_{1}/\left(m_{r}M\right)$, these equations may be re-written as:

$$\left(\omega_{1m}^{2}-\omega^{2}\right)y_{1}-\kappa_{m}y_{2}=0$$

$$\left(\omega_{1e}^{2}-\omega^{2}\right)y_{2}-\kappa \left(y_{1}+y_{3}\right)=0$$

$$\left(\omega_{1e}^{2}-\omega^{2}\right)y_{3}-\kappa \left(y_{2}+y_{4}\right)=0$$

$$\left(\omega_{1e}^{2}-\omega^{2}\right)y_{4}-\kappa \left(y_{3}+y_{5}\right)=0$$

(11) $$\left(\omega_{1m}^{2}-\omega^{2}\right)y_{5}-\kappa_{m}y_{4}=0,$$

For characteristic modes oscillating at the $\mu^{th}$ angular resonance frequency $\omega_{\mu }$, we may write $y_{\mu n}=Y_{\mu n}\;exp\left(j\omega_{\mu }t\right)$, where the constants $Y_{\mu n}$ define the overall mode shapes. The values $\omega_{\mu }^{2}$ are the eigenvalues of the tridiagonal matrix:

(12) $$\underline{M}=\left(\begin{array}{c} \begin{array}{ccc} \omega_{1m}^{2} & -\kappa_{m} & \begin{array}{ccc} 0 & 0 & 0 \end{array} \end{array}\\ \begin{array}{ccc} -\kappa & \omega_{1e}^{2} & \begin{array}{ccc} -\kappa & 0 & 0 \end{array} \end{array}\\ \begin{array}{c} \begin{array}{ccc} 0 & -\kappa & \begin{array}{ccc} \omega_{1e}^{2} & -\kappa & 0 \end{array} \end{array}\\ \begin{array}{ccc} 0 & 0 & \begin{array}{ccc} -\kappa & \omega_{1e}^{2} & -\kappa \end{array} \end{array}\\ \begin{array}{ccc} 0 & 0 & \begin{array}{ccc} 0 & -\kappa_{m} & \omega_{1m}^{2} \end{array} \end{array} \end{array} \end{array}\right),$$

When $\omega_{1m}^{2}$ is sufficiently different from $\omega_{1e}^{2}$, experience suggests that there will be little interaction between the outer masses and the remainder of the array. The eigenmodes will then separate into two groups. The first are the eigenvectors of the Toeplitz matrix ${\underline{M}}^{\prime }$, given by:

(13) $${\underline{M}}^{\prime }=\left(\begin{array}{ccc} \omega_{1e}^{2} & -\kappa & 0\\ -\kappa & \omega_{1e}^{2} & -\kappa \\ 0 & -\kappa & \omega_{1e}^{2} \end{array}\right)\;,$$

This matrix represents the dynamics of a reduced set of fully synchronized coupled resonators. Its eigenvalues are well-known, and lead to resonant frequencies:

(14) $$\omega_{\mu^{\prime }}=\sqrt{\left\{\omega_{1e}^{2}-2\kappa \cos\left(\frac{\mu^{\prime }\pi }{4}\right)\right\}},$$

Here $\mu^{\prime }=1,2,3$. The corresponding eigenvectors have the form:

(15) $$Y_{\mu^{\prime }n^{\prime }}=\sin\left(\frac{\mu^{\prime }n^{\prime }\pi }{4}\right),$$

Here $n^{\prime }=n-1$, and $n^{\prime }=1,2,3$. The second set contains symmetric and anti-symmetric modes that predominantly involve oscillation of the outer masses at $\omega_{1m}$. These responses may be damped using similar loads to the matched loads at the ports.

**Figure 4:**
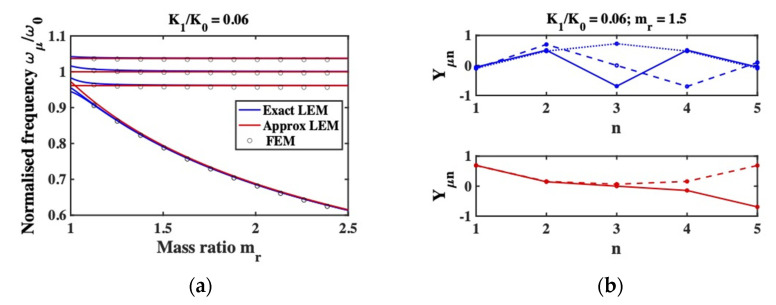
(**a**) Variation of exact and approximate resonant frequencies of a 5-beam system with mass ratio $m_{r}$, as predicted by the LEM for $K_{1}/K_{0}=0.06$. Points show predictions of a comparable FEM; (**b**) Exact mode shapes of a 5-beam system predominantly involving the inner (blue) and outer (red) beams, as predicted by the LEM for $K_{1}/K_{0}=0.06$ and $m_{r}=1.5$.

To confirm these arguments, the blue lines in Figure 4a shows the variation with $m_{r}$ of the exact resonant frequencies, for an array with example parameters $K_{1}/K_{0}=0.06$. The red lines show the approximate resonant frequencies. As $m_{r}$ rises, the exact solutions tend to the approximations, and resonances predominantly involving the outer masses separate from the remainder. Figure 4b shows the mode amplitudes $Y_{\mu n}$ for $K_{1}/K_{0}=0.06$ and $m_{r}=1.5$. The blue traces show the first set of modes while the red traces show the second. As predicted, the former is approximated by Equation (15), while the latter mainly involve the outer masses. Provided only the first modes are excited, the array behaves as if the outer masses are stationary, as shown in Figure 3b.

**Figure 5:**
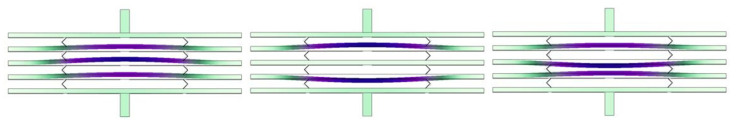
Exact mode shapes of a 5-beam system with motion predominantly involving the inner beams, as predicted by the FEM.

For comparison, the discrete points in Figure 4a show the predictions of the FEM for a 5-beam system with the same dimensional parameters as those used for Figure 2, which yields an equivalent value of $K_{1}/K_{0}$. A rectangular mass is added to beams 1 and 5, and the mass ratio is calculated as described above. The points follow the continuous traces almost exactly. Similarly, Figure 5 shows the shapes of the highest eigenmodes predicted by the FEM for $m_{r}=1.5$; the outer beams are almost stationary as expected.

## 4. Stiffness Matrix Model

In this section, we verify the previous arguments using the stiffness matrix method, which models beam networks by combining Euler beam bending theory with compatibility conditions [54,55]. The SMM is often preferred to FEM because of its increased speed. The high aspect ratio of most MEMS suspensions validates the use of Euler theory, and transducers may be modelled approximately as previously described [56,57].

### 4.1. Stiffness Matrix Model

Calculations were performed using a 2D SMM solver written in Matlab^®^ [61]. The stiffness matrix K was constructed from dimensions and material parameters, with $E_{0}$ reduced to model electrostatic detuning. Long beams were subdivided to ensure accuracy of resonant frequencies. Axial, transverse and angular displacements at each node were found for a vector of applied forces and torques (here a point load on the actuated beam).

Dynamic analysis was performed using additional mass and damping matrices. The mass matrix M was formed by combining dimensions and densities with standard relations for motions of centres of mass. The damping matrix C was modelled using Rayleigh’s method as R=aM+bK [60]. Here a and b are mass and spring damping coefficients, with a determined from the Q-factor and b=0. Ports were simulated by increasing the damping for these beams, using a damping coefficient determined from the load impedance $z_{L}$. Assuming harmonic forces and displacements as $\left(F,\;U\right)\;e^{j\omega t}$, substitution into the governing equation yields $\left(K-\omega^{2}M+j\omega R\right)U=F$. This equation was solved by inversion, and the velocity vector constructed as S=jωU. The scattering parameters $S_{11}$ and $S_{21}$ were then extracted from midpoint velocities.

### 4.2. Static Deflections

**Figure 6:**
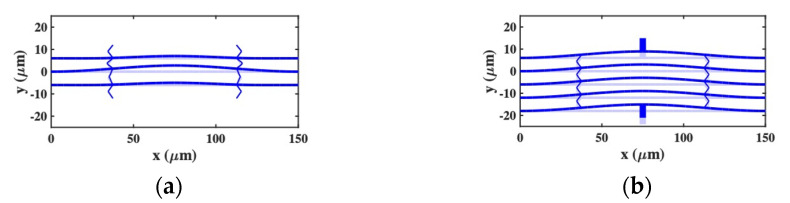
Deflection of coupled-beam systems with a DC bias applied, as predicted by the SMM: (**a**) 3-beam system with end-springs; (**b**) 5-beam system without end springs.

Responses were simulated for the same dimensional and material parameters as before. The single DC voltage $V_{D}$ and the impedances $z_{L}$ were adjusted to achieve tuning and matching at a design frequency of $f_{1}=1\;\mathrm{MHz}$, as $V_{D}=2.83\;V$ and $z_{L}=434\;k\Omega$, respectively. An AC voltage of $V_{A}=0.1\;\mathrm{mV}$ was again used for dynamic actuation. Figure 6a shows the magnified static deflection for a 3-beam system with end springs but no additional end beams, confirming that this design yields unequal deflection and hence unequal electrostatic tuning. Figure 6b shows the deflection for a 5-beam system equivalent to Figure 1b; the modified suspension clearly equalizes the deflection as required.

### 4.3. Frequency Responses

**Figure 7:**
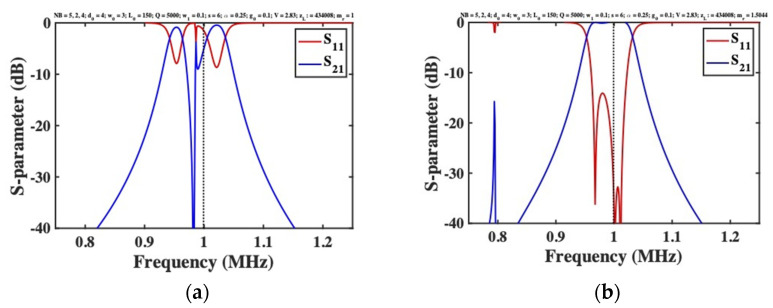
Frequency response of a 5-beam filter with electrostatic detuning (**a**) with and (**b**) without added mass, as predicted by the SMM.

Figure 7a shows the frequency variation of S-parameters for a 5-beam system with no added mass. As expected, the response is heavily distorted, with poor matching and a deep notch caused by the collective oscillation of an improperly synchronized system. Figure 7b shows the corresponding response when the first and fifth elements are loaded using masses with example width $5w_{0}$ and length s, yielding a mass ratio $m_{r}=1+\frac{5s}{\eta_{2}L_{0}}\approx 1.5$. Near the design frequency, the response is bandpass, with good matching and a flat passband. Additional unwanted transmission near 0.79 MHz can be attributed to excitation of the two low-frequency resonances in Figure 4.

**Figure 8:**
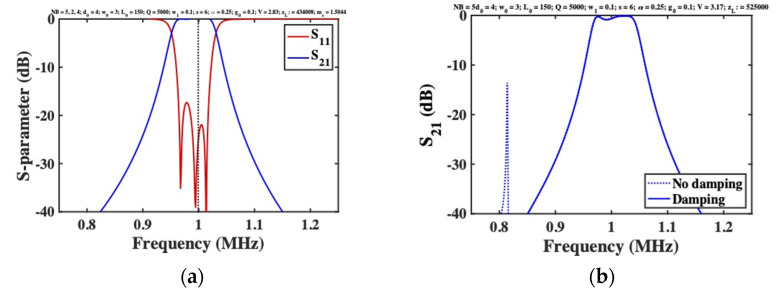
Frequency response of an electrostatically tuned 5-beam filter with added mass and additional damping of the end beams, as predicted by (**a**) the SMM, and (**b**) the FEM.

The unwanted response can be placed further out-of-band by increasing $m_{r}$; at the same time, passband flatness is improved. However, $m_{r}$ is limited by layout constraints. The dimensions of the mass may be reduced using material with density larger than silicon (for example, gold, with $\rho =19,300\;\mathrm{kg}/m^{3}$), but only at price of fabrication complexity. A simpler solution is to damp the unwanted response, by connecting loads in series with the DC bias for the first and last beams. Figure 8a shows the response obtained using loads identical to those used for matching. The unwanted resonances are suppressed, leaving the desired bandpass response unaltered and confirming the design strategy.

All the results presented here were compared with the predictions of the LEM, and excellent agreement was obtained in each case. The responses shown in Figure 7b and Figure 8a were also reproduced using the FEM. Figure 8b shows the frequency dependence of $S_{21}$ for a 5-beam system with mass-loaded end beams, obtained using COMSOL with and without end-beam damping. Apart from a minor difference in the tuning voltage (attributed to approximation of the transducer in the SMM), and minor passband ripple (which reduces with $m_{r}$ or $z_{L}$), the results are as expected. A bandpass response is obtained, and end-beam resonances are effectively suppressed by damping.

## 5. Conclusions

A method to compensate for the electrostatic desynchronization of coupled beam arrays has been proposed and confirmed by simulation, with excellent agreement being obtained using lumped element, stiffness matrix and finite element models. Additional beams are simply added at either end of the array, together with the coupling elements needed to ensure equal displacement of all beams under a DC tuning voltage. If the additional beams are mass-loaded, they take no part in the collective in-band response. The remainder of the array then exhibits properly synchronized behavior, and any out-of-band resonance may be suppressed using external loading. Since the additional mass need only place the unwanted resonances somewhere out-of-band, this arrangement is extremely tolerant to the exact mass value. The passband may then be adjusted in frequency with a single DC voltage, simplifying compensation for fabrication-induced variation of key nanoscale dimensions such as the width of the linking suspension or the electrode gap.

The combination of feature size and aspect ratio greatly complicates fabrication of any such device. To maximize the Q-factor, single crystal materials such as bonded silicon-on-insulator (BSOI) or silicon-on-glass wafers are preferred [8,11]. Mechanical parts may be formed by anisotropic plasma etching. However, advanced lithography such as the high-aspect-ratio combined poly and single crystal silicon (HARPSS) micromachining process would be needed to form sub-micron electrode gaps [43]. Sidewall transfer lithography (STL) has already been shown capable of integrating nanoscale suspensions with MEMS parts [45]. Electrical connection to fixed electrodes (many of which lie within the mechanical structure) also presents a challenge and is most easily achieved using buried or flip-chip bonded tracks.

The application presented here is to a three-beam filter. However, elimination of the need for multiple tuning voltages should simplify development of high-order filters based on larger beam arrays. Extension to four-port acoustic MEMS devices such as directional couplers and directional coupler filters (which allow more effective signal separation) is obvious, and we have already investigated example designs. Applicational to out-of-plane and torsional motion may also be possible. The principle is therefore general and may enable realization of a wide range of new filter devices.
